# Treatment With Remdesivir Alone or in Combination With GS‐441524 in Cats With Ocular Involvement of Feline Infectious Peritonitis: An Observational Case Series

**DOI:** 10.1111/jvim.70253

**Published:** 2025-10-01

**Authors:** Amy L. M. M. Andrews, Eneko Izaguirre, Jodie Green, Emma Scurrell, Savina Gogova, Sarah Tayler, Christiane Kafarnik

**Affiliations:** ^1^ Department of Clinical Science and Research, Royal Veterinary College Queen Mother Hospital for Animals, Ophthalmology Service Hatfield Hertfordshire UK; ^2^ Department of Clinical Science and Research Queen Mother Hospital for Animals, Internal Medicine Service, Royal Veterinary College Hatfield Hertfordshire UK; ^3^ Cytopath, Veterinary Pathology Ltd, Ledbury Herefordshire UK; ^4^ North Down Specialist Referrals, Bletchingley, United Kingdom (Previous Affiliation). Bristol Vet Specialists Ophthalmology Service Bristol UK

**Keywords:** anterior uveitis, cat, coronavirus, FIP, posterior uveitis, viral

## Abstract

**Background:**

Remdesivir and GS‐441524 have successfully treated cats with feline infectious peritonitis (FIP) but the changes in associated signs of ocular disease are not reported.

**Objective:**

Evaluate the response of FIP‐associated signs of ocular disease before and after treatment with remdesivir, GS‐441524, or both.

**Animals:**

Sixty‐one cats diagnosed with FIP by the Internal Medicine services between October 2021 and December 2022 that were treated with injectable, oral, or a combination of anti‐viral therapies were reviewed.

**Methods:**

Observation study. Signalment, associated signs of ocular disease, treatment, outcome, and follow‐up of affected cats were analyzed.

**Results:**

Associated signs of ocular disease in cats with FIP were diagnosed in 33% (20/61) cats, with a median follow‐up of 55 days (IQR 47.3–90.8, range: 16–1071 days) in 11 cats with FIP. Ocular changes were all associated with uveitis, of which 20% (4/20) had anterior uveitis, 25% (5/20) had posterior uveitis, and 55% (11/20) had panuveitis. Ocular disease occurred in effusive (11/20; 55%) and non‐effusive (9/20; 45%) FIP and was bilateral in 70% (14/20) cats. All cats started an 84‐day course of treatment, with the majority (17/20; 85%) receiving remdesivir in the initial period. Of those, 70% (12/17) received a high dose of 15–20 mg/kg/day. Eleven cats with uveitis had long‐term follow‐up nine (9/11; 82%) of these had resolution of uveitis. Eighty‐seven percent (13/15) of cases with anterior uveitis received topical anti‐inflammatory medication. Eighty percent (16/20) of cats survived.

**Conclusion and Clinical Importance:**

Cats with FIP commonly have ocular involvement. Associated uveitis responded to remdesivir or GS‐441524 treatment effectively in 82% of cases.

**Abbreviations**
BSH: British short hair
CSF: cerebrospinal fluid
DSH: domestic short hair
FCoV: feline enteric coronavirus
FIP: feline infectious peritonitis
IOP: intraocular pressure
NSAID: non‐steroidal anti‐inflammatory drugs
RNA: ribonucleic acid
RT‐PCR: reverse transcription polymerase chain reaction
RVC: Royal Veterinary College

## 1 Introduction

Feline infectious peritonitis (FIP) is a widespread disease caused by a highly pathogenic mutant of the ubiquitous feline enteric coronavirus (FCoV). Historically, no effective treatment has been available. However, in recent years, multiple studies describe antiviral compounds that inhibit viral ribonucleic acid (RNA) synthesis with promising outcomes [1, 2, 3, 4, 5, 6].

FIP can be classified as effusive (wet) or non‐effusive (dry), with or without ocular or neurological involvement [5]. Whilst clinical classification guides diagnostics and treatment, the histopathological overlap, such as the presence of pyogranulomas identified on post‐mortem examination in both non‐effusive and effusive cats, suggests these forms are not distinct disease entities [7, 8]. Ocular and neurological involvement occur more frequently in cats with non‐effusive FIP and are documented in up to 36% of cases [7, 8, 9, 10]. Less often, ocular involvement occurs in up to 5% of effusive cases of FIP [8, 10].

Ocular manifestations of FIP include anterior or posterior uveitis with examination findings such as keratic precipitates, dyscoria, anisocoria, change in iris color, hyphema, hypopyon, and fibrinous exudate [8, 9, 11]. Posterior segment abnormalities include hyporeflective tapetal lesions, gray to white non‐tapetal lesions, retinal hemorrhage, tortuosity of retinal vasculature, detachment, and perivascular effusions [8, 9]. There is an “ocular‐signs dominant” form of FIP, in which uveitis is the most commonly observed clinical feature [12].

Antiviral treatment of FIP consists of the use of GS‐441524 with or without prior administration of its prodrug remdesivir (GS‐5734) [1, 2, 4, 5, 13, 14]. GS‐441524 is a nucleoside analogue that competes with endogenous nucleoside pools, preventing viral RNA replication by causing premature termination of viral RNA synthesis, therefore, preventing viral transcription and translation [15]. Remdesivir improves cellular penetration of the parent nucleoside GS‐441524 [15]. Before the use of antiviral medication, the prognosis for cats with FIP was grave, with uveitis remaining persistent despite supportive anti‐inflammatory drugs [16].

There is limited information on cats with FIP with ocular involvement having undergone treatment with remdesivir or GS‐441524 [1, 2, 3, 4, 5, 13, 17]. In experimentally infected cats treated with GS‐441524, 22%–33% of the plasma concentration was detected in the aqueous humor [4]. Resolution of signs of ocular disease occurs in cats with naturally occurring FIP under treatment with GS‐441524 [2]. Absence of FCoV antigen in numerous tissue samples from around the body and the aqueous humor 164 days after the treatment course of GS‐441524 is documented [13]. Dose ranges for the use of remdesivir and GS‐441524 are variable, with higher doses of up to 20 mg/kg being recommended in cases with ocular or neurologic involvement [18].

The aim of this observational case series was to describe presenting signs of ocular disease, treatment protocols, clinical progression, and outcomes of cats with FIP and ocular involvement treated with remdesivir, GS‐441524, or a combination of both.

## 2 Materials and Methods

This observational study was carried out at the Queen Mother Hospital for Animals (QMHA) and North Down Specialist Referrals (NDSR) recruiting cats from October 2021 to December 2022 (ethical approval URN: amended SS2021‐0208).

All cats diagnosed with FIP underwent an ophthalmic examination including tonometry, slit lamp biomicroscopy and posterior segment examination including fundoscopy. Clinical diagnosis of concurrent ocular disease in cats diagnosed with FIP was made by a diplomate of the European College of Veterinary Ophthalmologists or a resident working under their direct supervision. All cats included had been treated with either remdesivir, GS‐441524 or a combination of both, for a period of 84 consecutive days (unless the cat died or was humanly euthanized before completion of the course) [5]. If active signs of uveitis consistent with FIP were identified, then the cat was included in the current study. Each cat that was diagnosed with ocular involvement underwent ophthalmic examination daily during hospitalization. After discharge, cats with FIP‐associated signs of uveitis underwent ophthalmic examination at every subsequent consultation at the QMHA. The case diagnosed at NDSR was recruited solely by an author due to the specific histopathologic findings in that cat. Any cat treated with medication not sourced from a veterinary‐approved pharmaceutical manufacturer was excluded due to legal considerations and uncertainty regarding drug composition [12, 19].

### 2.1 Signalment and Presenting Clinical Signs

Data recorded included sex and neuter status, age (in months) at the time of presentation, breed, days from admission to starting antiviral medication, presence or absence of concurrent signs of neurological disease, diagnosis of effusive or non‐effusive form of FIP and the number of days of hospitalization. The findings of the ocular examination were classified as anterior, posterior and panuveitis. Secondary glaucoma was defined as an intraocular pressure (IOP) > 25 mmHg [20]. Ocular hypotension was classified as < 10 mmHg and notably (> 20%) lower intraocular pressure than the normal eye, if there was unilateral disease [21].

### 2.2 Diagnosis of FIP

Clinical diagnosis of FIP was carried out by a board certified diplomate of Internal Medicine or a resident under their direct supervision, based on a combination of signalment, clinical signs, hematologic and serum biochemistry findings, imaging, coronavirus reverse transcription polymerase chain reaction (RT‐PCR) in cerebrospinal fluid (CSF) and cytologic examination as previously published [5, 8]. Coronavirus RT‐PCR on effusions, aqueous humor or lymph node aspirates, immunocytochemistry and immunohistochemistry were considered desirable but not essential for diagnosis if there was a very high index of suspicion for the disease based upon other clinicopathologic findings. Details of the results of selected hematology/biochemistry values and further diagnostic tests for each cat can be found in Table S1 of the supplementary information.

### 2.3 Treatment of FIP

The information regarding the therapeutic regime was recorded as: dose, route of administration (injectable, oral), duration (days) of remdesivir and GS‐441524; duration of topical ocular medication, concurrent systemic anti‐inflammatory medication; concurrent oral administration of gabapentin or local anesthetic administered topically (EMLA 5%, AstraZeneca) during subcutaneous treatment. Concurrent topical and systemic anti‐inflammatory treatment, topical cycloplegic, anti‐glaucoma medication, and topical antibiotic treatment were prescribed at the discretion of the ophthalmologist or resident under supervision. The decision regarding choice and route of administration of antiviral medication was at the discretion of the attending internal medicine clinician, however, was largely based upon the ability to administer oral medications and availability of GS‐441524 suitable for oral administration, as at the beginning of case recruitment there was only legal access to injectable remdesivir necessitating some cases to receive subcutaneous injections. The initial treatment phase was defined as the treatment received during hospitalization followed by the continuing treatment after discharge to complete the treatment course of 84 days [5].

### 2.4 Follow‐Up

Follow‐up information was recorded to the last ophthalmological re‐examination in days after starting the 84‐day treatment course, ophthalmic examination findings, ocular topical and systemic anti‐inflammatory treatment and duration, cause for enucleation and reason for euthanasia. Short‐term follow‐up was defined as the period from initial presentation to the time of discharge (after clinical improvement once antiviral therapy was initiated). Long‐term follow‐up referred to any continued monitoring or reassessment beyond this initial discharge period.

### 2.5 Histopathological Analysis and Coronavirus RT‐PCR

Two eyes were enucleated, one from each cat, of which one aqueous humor sample was tested for feline coronavirus viral RNA by RT‐PCR (Veterinary Pathology Group, Exeter, United Kingdom). The remaining globe was transferred into a 10% neutral‐buffered formalin. The sample was submitted for histopathological analysis. The fixed sample underwent tissue processing, paraffin embedding, sectioning at 5 μm slice thickness, and hematoxylin–eosin staining.

### 2.6 Statistical Analysis

Basic descriptive statistics were recorded, manually reviewed and analyzed using Microsoft Excel (Microsoft Corporation, Redmond, WA, USA, 2022). Categorical data were summarized with count and percentage. Data with a normal distribution were reported as mean ± standard deviation, whereas skewed data were presented as median with interquartile range (IQR) and range.

## 3 Results

A total of 61 cats were diagnosed with FIP during the 14‐month recruitment period by a diplomate of the European or American College of Veterinary Internal Medicine or a resident working under their supervision, of which 60 presented to the QMHA Internal Medicine or Ophthalmology service and one cat to the Ophthalmology service at North Down Specialist Referrals [5].

### 3.1 Signalment

Twenty out of 61 FIP cases (20/61; 33%) presented with signs of ocular disease (Table S1 of supplementary information). Four of these cats (4/20; 20%) were also included in a previous case series [5]. Three‐quarters of the study sample were purebred cats (15/20; 75%), composed of six British Short Hair (BSH), three Ragdoll, two Maine Coon one Burmese, one Turkish Van, one Sphynx, and one Siberian cat. The remaining five cats were Domestic Short Hair (DSH). Over two‐thirds of the study sample were male cats (14/20; 70%; seven entire males, six neutered males) and four entire females and three spayed females. Median age at presentation was 11 months (IQR 6–19.5, range: 1–60).

### 3.2 Clinical Presentation

The majority of cats were primarily referred to the Internal Medicine service via the Emergency and Critical Care service; two cats were referred primarily for ocular abnormalities (2/20; 10%). Out of 20 FIP cats with associated signs of ocular disease, 11 presented with the effusive form of FIP (11/20; 55%), and 9 (9/20; 45%) with the non‐effusive form.

The majority of cases had bilateral ocular disease (14/20; 70%) and over half presented with signs of panuveitis (11/20; 55%), four cases with clinical signs of anterior uveitis only (4/20; 20%), and five with signs of posterior uveitis only (5/20; 25%; Table 1). Four of the cases presented with or developed secondary glaucoma (4/20; 20%). Concurrent FIP‐associated signs of neurological disease were found in five cases (5/20; 25%).

**TABLE 1:** Classification criteria and ophthalmic signs in feline infectious peritonitis cases presenting with anterior and posterior uveitis.

Ocular abnormalities	Number of cases (n/20)	N = %
Clinical signs of anterior uveitis	15	75
Intraocular hypotension (< 10 mmHg)	12	60
Aqueous humor flare	11	55
Keratic precipitates	7	35
Rubeosis iridis	5	25
Fibrin in the anterior chamber	3	15
Anisocoria	3	15
Iris swelling	2	10
Hyphema	2	10
Iris color change	1	5
Clinical signs of posterior uveitis	16	80
Hyporeflective lesions of the tapetal fundus/gray to white lesions of the non‐tapetal fundus	14	70
Retinal detachment	2	10
Retinal hemorrhage	1	5
Hyalitis	1	5
Vitreal hemorrhage	1	5
Clinical signs of panuveitis	11	55
Secondary glaucoma	4	20

### 3.3 Treatment

#### 3.3.1 Antiviral Treatment

**FIGURE 1:**
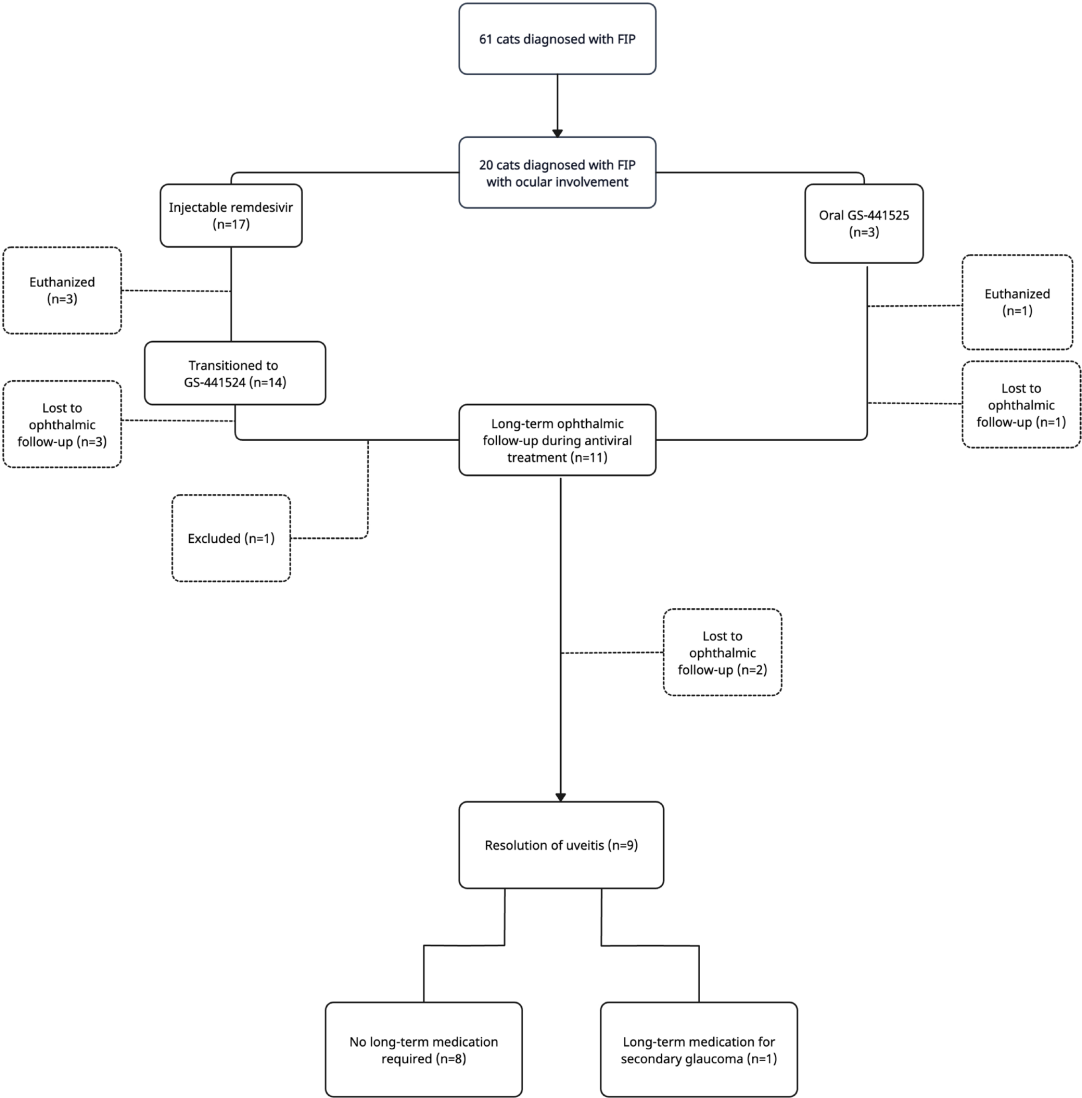
Flow diagram illustrating the different treatment courses and outcomes with injectable remdesivir (15–20 mg/kg; 10–14 mg/kg) and oral GS‐441524 (15–22 mg/kg; 10–14 mg/kg) for 20 cats diagnosed with feline infectious peritonitis (FIP) with ocular involvement.

Initial treatment in 17 cats (17/20; 85%) with FIP‐associated signs of ocular disease was injectable remdesivir (SC or IV), whereas three cats (3/20; 15%) received only GS‐441524 (16–19 mg/kg) orally. Over half (12/20; 71%) of the cats received a dose of 15–20 mg/kg of remdesivir, and two cats (2/20; 10%) started at a lower dose of 10–14 mg/kg of remdesivir for a median of 5 days (IQR 3–6, range: 3–84 days) [5]. Three cats (3/20; 15%) were euthanized before transitioning to GS‐441524 orally, two (2/3; 66%) of which had received a 10–13 mg/kg dose of remdesivir previously the remaining cat (1/3; 33%) received a 20 mg/kg dose (Figure 1).

Seventeen cats received GS‐441524 (17/20; 85%) for a median of 79 days (IQR 74.5–81, range: 0–84 days). Fourteen cats (14/17; 82%) received the higher dose 15–22 mg/kg, and three cats (3/17; 18%) received GS‐441524 administered orally at the lower dose (10–14 mg/kg). One cat receiving the higher dose of GS‐441524 orally was euthanized the evening after discharge from the hospital due to suspected gastric hemorrhage.

#### 3.3.2 Other Concurrent Treatment

Meloxicam was administered to six cats (6/20; 30%). Of these, one cat received it for 3 days before euthanasia due to suspected gastric hemorrhage, and in two cats, meloxicam was discontinued due to the development of diarrhea. Two further cats received meloxicam; however, this was discontinued by the owner due to difficulties medicating at home. The remaining cats received meloxicam for a total of 53 and 98 days. Robenacoxib (1/20; 5%) was received by one cat for 12 days due to the onset of diarrhea after one dose of meloxicam. Meloxicam was administered for a median duration of 22 days (IQR 16–53, range: 4–98 days). A 14‐day course of prednisolone was administered orally to one cat (1/20; 5%) another cat received prednisolone systemically, however, it was later excluded from the study. Topical anti‐inflammatory (13/15; 87%) and cycloplegic (9/15; 60%) treatment atropine (Minims Atropine Sulphate 1%), cyclopentolate (Minims Cyclopentolate Hydrochloride 0.5%, or tropicamide (Mydriacyl 1%)) was initiated in cats presenting with anterior/panuveitis and was administered for a median of 43 (IQR 8–81, range 1–1071) and 20.5 days (IQR 13.25–21.25, range 1–41), respectively. Four cases with glaucomatous disease were treated with carbonic anhydrase inhibitors (4/15; 27%; Table 2). Three cats developed a superficial corneal ulcer and received chloramphenicol eye drops (3/20; 15%). One cat (1/20; 5%) received gabapentin orally and local anesthetic topically (EMLA 5%, AstraZeneca) due to discomfort associated with SC treatment of remdesivir.

**TABLE 2:** Concurrent topical medication feline infectious peritonitis cats presenting with signs of anterior/panuveitis.

Topical ophthalmic medication	Number of cases n/15 (%)
Ketorolac (Acular)	8 (53)
Bromfenac (Yellox)	3 (20)
Prednisolone acetate (PredForte)	3 (20)
Dexamethasone (Maxidex)	1 (7)
Flurbiprofen (Ocufen)	1 (7)
Cyclopentolate (Minims Cyclopentolate Hydrochloride 0.5%)	5 (33)
Atropine (Minims Atropine Sulphate 1%)	3 (20)
Tropicamide (Mydriacyl 1%)	1 (7)
Brinzolamide (Azopt)	3 (20)
Dorzolamide (Trusopt)	1 (7)

### 3.4 Outcome

Of the 16 (16/20; 80%) surviving cats, 11 included cases (11/20; 55%) that had long‐term follow‐up (Figure 1). The median time of follow‐up was 55 days (IQR 47.3–90.8, range: 16–1071 days) after starting treatment with remdesivir or GS‐441524. Six cats (6/20; 35%) were lost to ophthalmic follow‐up, however, no clinical signs suggestive of ocular disease were raised with the Internal Medicine service during ongoing verbal communications. Two cases with uveitis had uncontrolled secondary glaucoma and underwent unilateral enucleation (2/20; 10%). Four cases (4/20; 20%) were euthanized due to FIP‐associated clinical signs after a median of 1.5 days from commencing treatment with antiviral medication (IQR 1–3.75, range: 1–6 days). Two of the cats (2/20; 10%) were euthanized due to deterioration in systemic health, one cat was euthanized due to worsening neurological status (1/20; 5%) and the remaining cat (1/20; 5%) suffered suspected gastric hemorrhage and was euthanized. One case (1/20; 5%) with persistent uveitis was excluded during long‐term follow‐up due to sourcing medication from a non‐licensed supplier.

**FIGURE 2:**
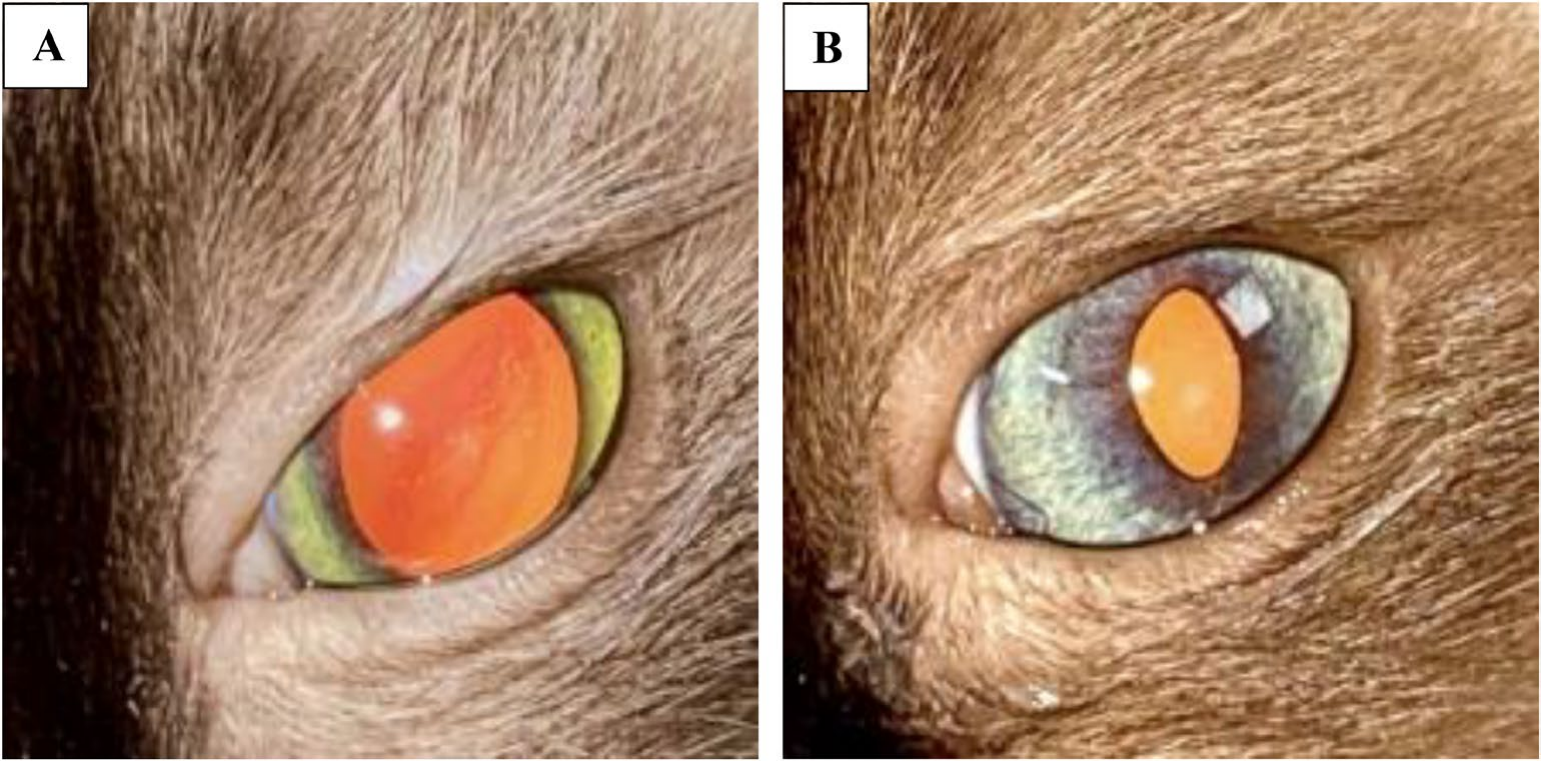
A 6‐month‐old, female entire, Ragdoll diagnosed with effusive FIP with unilateral anterior uveitis. Image A shows iris color change of the left eye (mydriatic agent had been applied). Image B shows marked improvement in iris color change after 3 days of remdesivir administered intravenously.

**FIGURE 3:**
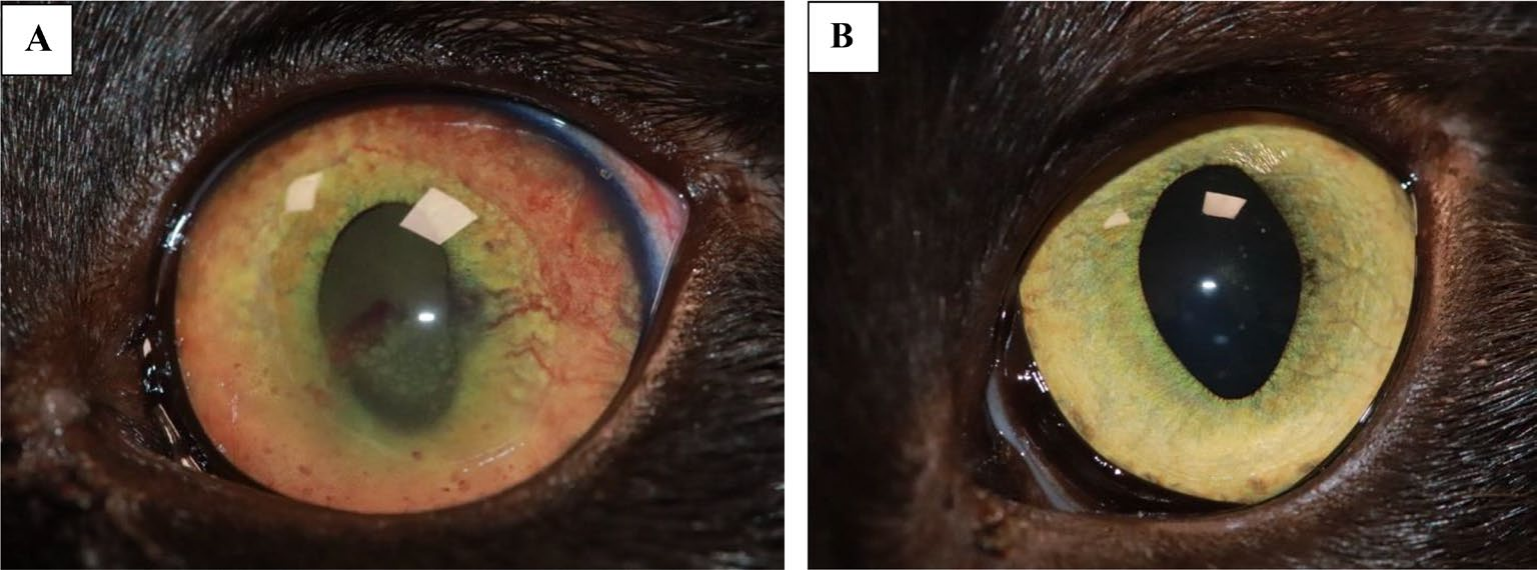
A 2‐year‐old, male neutered, domestic short hair diagnosed with non‐effusive FIP with bilateral panuveitis. Image A shows hyphema, fibrinous uveitis, rubeosis iridis, and keratic precipitates before treatment. Image B shows resolution of uveitis 3 months after completion of an 84‐day course of antiviral medication.

**FIGURE 4:**
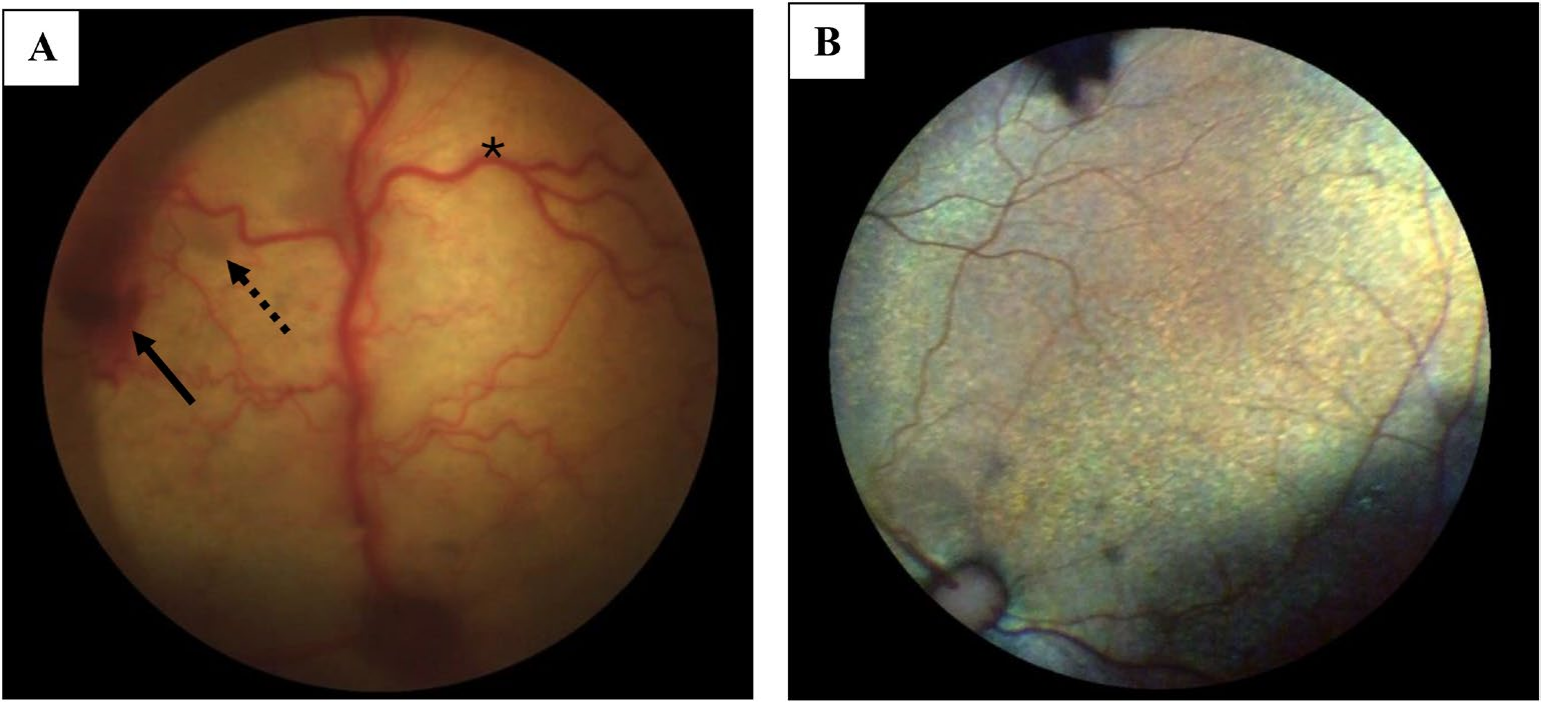
A 9‐month‐old, male neutered, Burmese with non‐effusive FIP with bilateral posterior uveitis. Image A shows multifocal regions of retinal hemorrhage (black arrow) and hyporeflective tapetal lesions (dashed arrow) with tortuosity of the retinal vessels (asterisk) on the first day of antiviral treatment. Image B shows resolution of active posterior uveitis 3 days after completion of an 84‐day course of antiviral medication.

**FIGURE 5:**
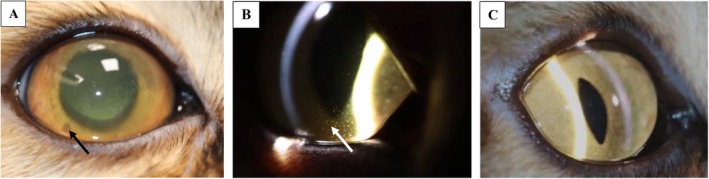
A 1 year 6‐month‐old, male neutered, Siberian with non‐effusive FIP presented with bilateral asymmetrical anterior uveitis. Image A shows mutton fat precipitates (black arrow) and rubeosis iridis before antiviral treatment (mydriatic agent had been applied). Image B resolution of rubeosis iridis on day 22 of treatment, keratic precipitates remained (white arrow). Image C shows resolution of keratic precipitates 2.5 months after completion of an 84‐day course of antiviral medication. The cat remains on long‐term management for unilateral secondary glaucoma and sub‐trace aqueous humor flare in the right eye.

**FIGURE 6:**
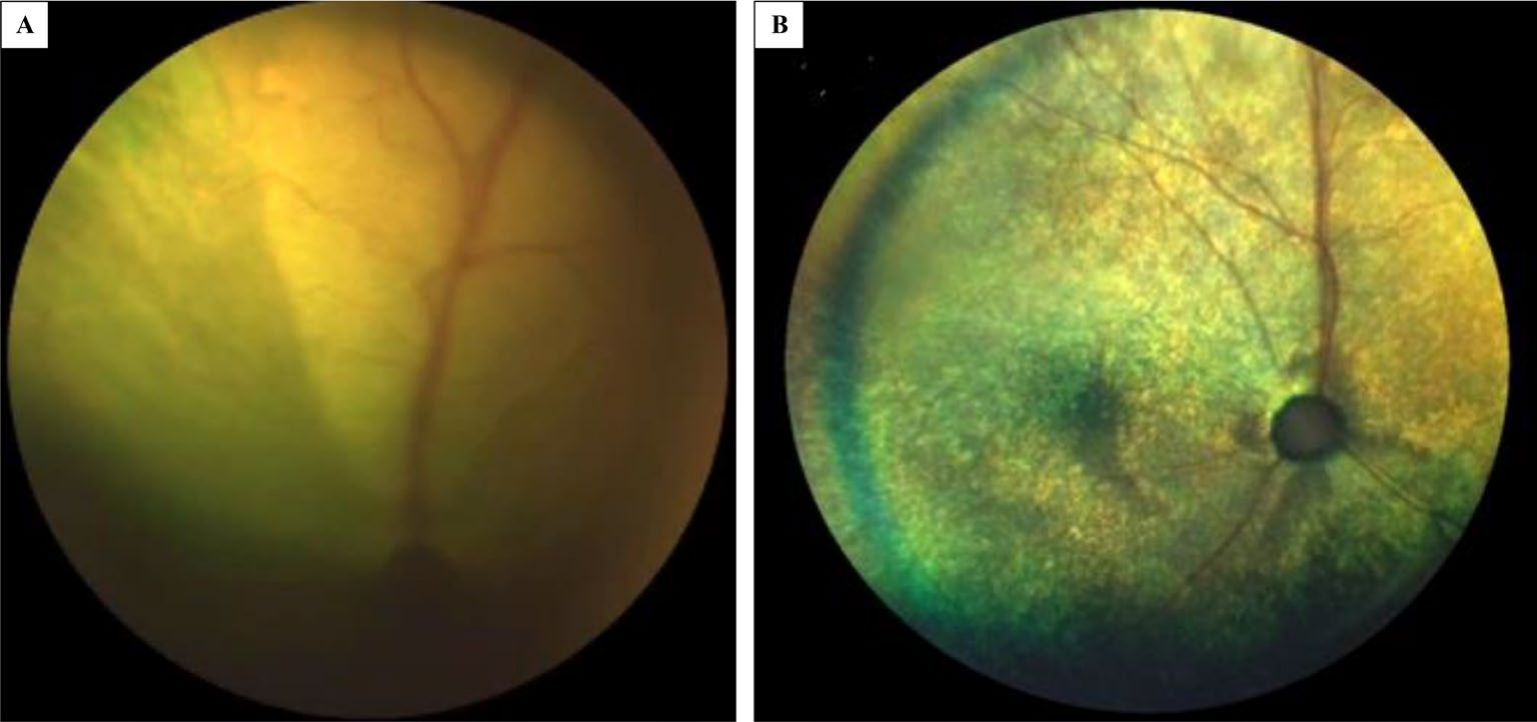
A 1‐year 5‐month‐old female neutered British Short Hair with non‐effusive FIP presented with vision loss. Image A shows retinal edema and a ventromedial retinal detachment. Image B shows reattachment of the retina 4 months after the course of antiviral medication; some regions of tapetal hyperreflectivity remained.

Clinical resolution of the uveitis (determined by the lack of aqueous humor flare and hyporeflective fundic tapetal lesions) was reported in nine cats (9/11; 82%) after a median duration of 44 days (IQR 32–52.5, range 20–71) of antiviral medication, and eight cats (8/11; 73%) were no longer receiving topical or systemic medication at the time of the last follow‐up (Figures 2, 3, and 4). One cat (1/11; 9%) had resolution of uveitis of the right eye at day 63; however, persistent low‐grade anterior uveitis of the left eye remained, this cat was later lost to follow‐up. One cat (1/11; 9%) received antiviral treatment only and had complete resolution of FIP‐associated ocular disease. One cat (1/11; 9%) had secondary glaucoma identified on day 24 of antiviral treatment and received brinzolamide for 35 days before the glaucoma resolved. One cat with documented glaucoma (1/11; 9%) continues to be managed with brinzolamide (Azopt) and bromfenac (Yellox) 3 years later and undergoes regular intraocular pressure curves despite resolution of uveitis (Figure 5, cat 5). The case with vision loss associated with retinal detachment (Figure 6A) regained vision after retinal reattachment (Figure 6B) once antiviral medication and systemic meloxicam had been initiated (Table S1, cat 9).

### 3.5 Coronavirus PCR and Histopathology

**FIGURE 7:**
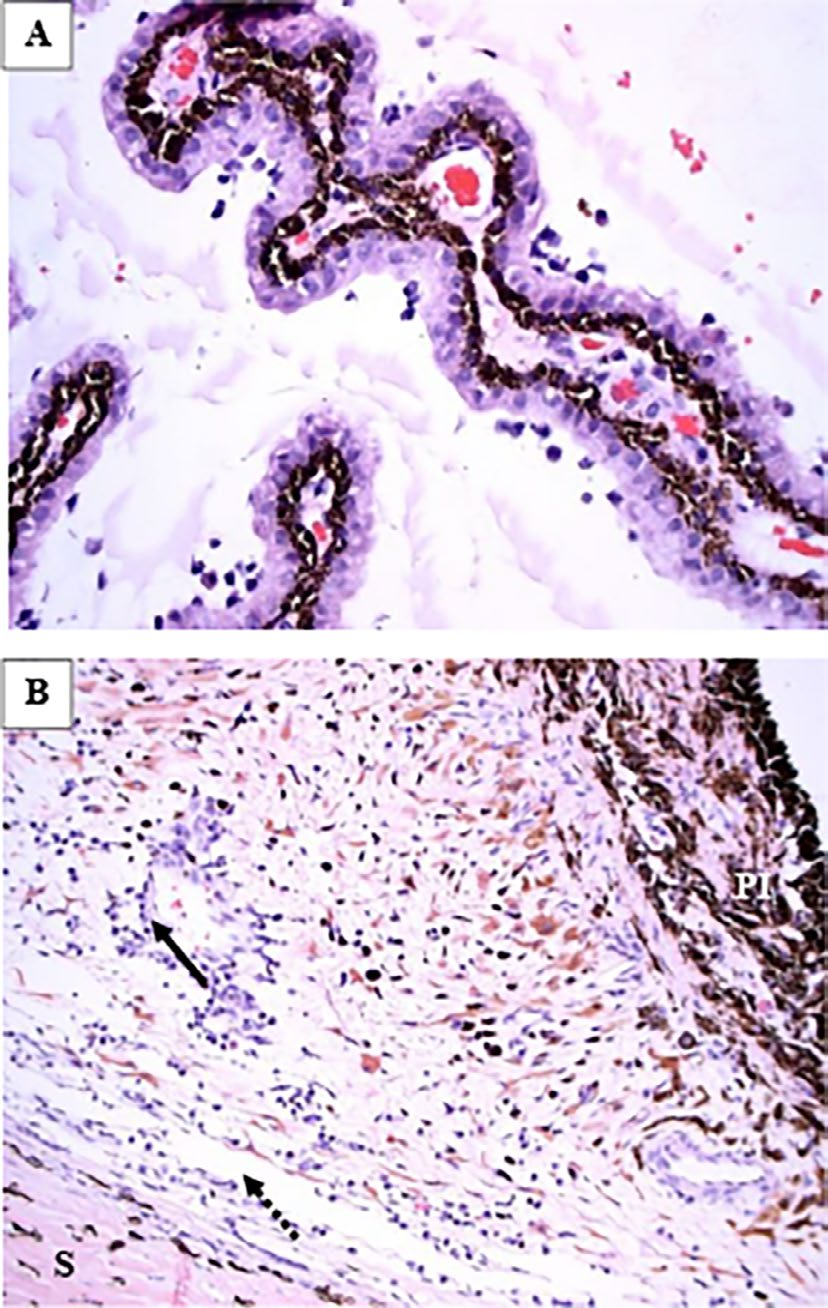
Histopathology image of the ciliary process of an enucleated globe with uveitis and glaucoma revealed coating of lymphocytes and plasma cells: Hematoxylin and eosin stain. 400 × magnification (A). Histopathology image of the iris base with mild perivascular lymphoplasmacytic inflammation (black arrow) and a collapsed trabecular meshwork (dashed arrow). S, sclera, PI, posterior aspect of the iris: Hematoxylin and eosin stain. 200 × magnification (B).

One cat (1/20; 5%) required enucleation of one eye due to unresponsive secondary glaucoma 106 days after having completed its 84‐day course of antiviral treatment (14 days of remdesivir administered subcutaneously at 20 mg/kg followed by 70 days of GS‐441524 administered orally) and remained free of FCoV in the aqueous humor. Histopathology results revealed mild to moderate lymphoplasmacytic anterior uveitis (Figure 7A), mild intermediate uveitis associated with pre‐iridal inflammatory membranes and peripheral anterior synechiae with collapse of the ciliary cleft and trabecular meshwork (Figure 7B), focal posterior cortical cataract, and inner retinal atrophy with ganglion cell loss most marked in the non‐tapetal retina.

Histopathology was obtained for a second cat that had received 5 days of remdesivir (20 mg/kg dose) followed by GS‐441524 orally. On day 12 of treatment, the eye was enucleated due to refractory glaucoma and persistent uveitis. Histopathology showed a mixed inflammatory population within the anterior chamber consisting of eosinophils, neutrophils, and foamy macrophages. A pre‐iridal fibrovascular membrane spanned across the iridocorneal angle and was infiltrated with plasma cells, lymphocytes, and macrophages retinal ganglion cell loss was also identified.

One cat that was euthanized (1/4; 25%) underwent post‐mortem examination and histopathological findings were consistent with FIP.

## 4 Discussion

This observational case series provides detailed ophthalmic data and follow‐up findings on cats with FIP treated with the antiviral agents remdesivir and GS‐441524. These results add to the limited peer‐reviewed literature, which currently consists mainly of case reports reporting the ocular response to treatment in such cases.

In the present study, 80% (16/20) of cats diagnosed with FIP with ocular involvement survived and recovered with dramatic improvement of the associated signs of ocular disease during the treatment period of 84 days. The systemic follow‐up of these cases is beyond the scope of the current study [5]. Nine cases (9/11; 82%) with ophthalmic follow‐up information remained without signs of clinical uveitis within the follow‐up time, with only one cat requiring long‐term topical medication for associated secondary glaucoma. One case with follow‐up (1/11; 9%) received antiviral treatment only and had complete clinical resolution of the associated ocular disease without topical or systemic anti‐inflammatory treatment; this management approach could be considered. It is unknown whether this confers a long‐term risk of developing secondary glaucoma due to long periods of low‐grade, subclinical uveitis. Overall, these are encouraging results with the limitation of this being a small case series, but they demonstrate the high efficacy of remdesivir and GS‐441524 for the treatment of naturally occurring FIP with ocular involvement, in line with previously published studies [1, 2, 4, 5].

The higher incidence of FIP in younger purebred cats has been repeatedly reported in the literature and was confirmed by the present case series, with three quarters being purebred and young cats (15/20; 75%) [1, 11, 22]. Proposed explanations include enhanced susceptibility or resistance in highly inbred purebred populations and increased population density in purebred catteries [17]. There were more male cats affected than females which has also been consistently reported in the literature and has been attributed to their behavioral traits [17, 23, 24]. The percentage of FIP cats with ocular involvement in the current study (20/61; 33%) was similar to previous reports of 29% (25/86) [7]. This demonstrates the importance of performing a full ophthalmic examination in any cat for which FIP is suspected [7].

Uveitis is most commonly (60%) identified in the non‐effusive form of FIP and with less than 5% of effusive cases of FIP developing ocular involvement [8, 10, 11]. Contrary to these findings, the present case series presented with over half of cases (11/20; 55%) being affected by the effusive form of FIP. This could be explained by the implications of the disease's historical grave prognosis, particularly in cases exhibiting more “classic” clinical signs of thoracic or abdominal cavity effusions. In such instances, the incubation period of the disease can be shorter, therefore, the urgency of the clinical situation might have led to a reduced emphasis on comprehensive ophthalmic examinations [11]. This oversight could contribute to the discrepancies observed in previous literature, as the potential ocular manifestations of FIP were not fully explored in affected cats.

The initial subjective clinical observation that the majority of FIP cases with signs of ocular disease also present with signs of neurological disease could not be confirmed by this case series [25]. Only a quarter of cases with signs of ocular disease also had neurological disease (5/20; 25%), similar to other reports [2, 25]. Four out of five (4/5; 80%) cats that presented with ocular and neurological disease treated with higher doses of remdesivir, GS‐441524, or a combination of the two medications, recovered fully [5].

Treatment protocols in this study were based on dosages used in human medicine and previous veterinary studies on the dosing of GS‐441524 [1, 2, 4, 5, 13, 26, 27]. Optimal dosing for GS‐441524 administered orally is also yet to be established, however, doses of 10–20 mg/kg/day for 84 days have been used with successful results which is higher than the doses used in initial studies (2–10 mg/kg) [1, 2, 4, 5, 13, 27]. Splitting the daily dose of GS‐441524 into twice daily could result in optimized GS‐441524 serum concentrations [28]. A shorter duration treatment protocol of 42 days has promising results [24]. Several preliminary studies suggest that ocular and neurological forms of FIP require higher dosages due to limited drug penetration through the blood‐ocular and blood–brain barriers [1, 2, 4, 25].

There are reports that all cats with the neurological form of FIP have ocular involvement; but, only a quarter of cats (5/20; 25%) in our study exhibited signs of both forms, similar to other reports, although there are reports of concurrent ocular and neurological involvement in only 3% of FIP cases [1, 2, 12]. These findings highlight the considerable variability in the co‐occurrence of ocular and neurological manifestations, likely influenced by the immune‐privileged status of these sites and the unpredictable nature of FIP progression. Aqueous humor concentrations of GS‐441524 reach 22%–33% of plasma levels [4]. This, in combination with clinical experience during the study period, resulted in most of the FIP cases with ocular involvement being administered dosages of 15 to 20 mg/kg remdesivir or GS‐441524 once daily [5]. All cases were administered remdesivir exclusively at the beginning of the case collection period as GS‐441524 was not yet available in an oral preparation. GS‐441524 became available as an oral preparation in November 2021 and became the initial antiviral therapy in appetent cats [5]. Two of the four FIP cats which were euthanized before finishing the 84 days treatment course presented with signs of systemic disease, which were too advanced to draw a conclusion on dosing regime, but were initially treated with lower doses of remdesivir.

Clinical resolution of secondary uveitis was reported in nine cats (9/11; 82%) that had follow‐up ophthalmic examinations. All but one cat had discontinuation of topical and systemic anti‐inflammatory medication, suggesting that the concurrent inflammatory stimulation had resolved. The histopathology of an enucleated eye reported the absence of pyogranulomatous inflammation, perivasculitis, or proteinaceous effusion to support active FIP infection [13, 29]. An aqueous humor sample from the same globe was obtained at the time of enucleation which demonstrated absence of feline coronavirus RNA by RT‐PCR. These results suggest complete resolution of ocular involvement. A pre‐treatment sample of the aqueous humor, however, was not obtained, so the presence of viral RNA in the aqueous humor before commencement of treatment cannot be ascertained. FCoV RNA can be detected in blood before treatment, blood viral loads decrease by day two to four after treatment initiation and by day 14, viral RNA is no longer detected [13]. Whether this holds true for aqueous humor viral RNA remains unknown.

Associated secondary uveitis responded to remdesivir or GS‐441524 treatment effectively in the short‐term in most of these cases. However, the median follow‐up time in the present case series was 55 days, therefore, long‐term ocular follow‐up is needed to draw further conclusions on failures.

In the initial study sample, four (4/20; 20%) cats presented with glaucoma secondary to uveitis. Of these, two of the cats that completed the antiviral course required unilateral enucleation due to uncontrolled glaucoma. One cat received brinzolamide for 35 days until glaucoma resolved, and the other cat remains controlled on anti‐glaucoma medication (brinzolamide, Azopt) and had resolution of uveitis long‐term. Clinicians should be vigilant for uveitis‐associated secondary glaucoma and perform tonometry at regular intervals, particularly in uveitic eyes which have inappropriate normal to high‐normal intraocular pressure. Carbonic anhydrase inhibitors are the topical treatment of choice for cats with secondary glaucoma, and steroid‐induced ocular hypertension should be considered as a potential contributing factor [30, 31].

Systemic antiviral medication as sole treatment of uveitis would be of great interest for future studies. One cat that presented with panuveitis received antiviral treatment as a sole therapy and showed a rapid response to treatment with complete resolution of signs of ocular disease within the treatment course. Five further cats received antiviral treatment as a sole therapy but were lost to follow‐up, so definitive resolution cannot be assumed; however, no ocular concerns were reported by their owners during verbal communications with the Internal Medicine service. Although a combination of topical and systemic treatment was used in most cases, and this could be a confounding factor, both oral and topical anti‐inflammatory medication were discontinued in most cases without reported relapse in clinical signs, suggesting that the concurrent inflammatory stimulation and response were resolved. The choice of additional systemic NSAIDs or glucocorticoids was based on the presence of pan‐ and posterior uveitis but was rejected by the Internal Medicine service when there were concerns for hypotension and increased chances of gastrointestinal hemorrhage. One of the four cats that did not complete the full course during initial antiviral treatment suffered suspected gastric hemorrhage and death after 3 days of administration of meloxicam for the treatment of posterior uveitis. Therefore, the clinical benefit of systemic anti‐inflammatory medication should be considered as the pharmacokinetics of this drug in combination with antiviral therapy in cats with FIP is currently unknown. They should be used in caution particularly in critical cases in which there could be periods of gut hypoperfusion that could contribute to NSAID or glucocorticoid‐related gastrointestinal hemorrhage [18, 32].

This study's main limitations were associated with its observational nature and the small number of cases. Diagnosing FIP can be challenging; therefore, it is possible that some cats included in this study were misdiagnosed. However, as diagnoses were made in accordance with recognized guidelines, the likelihood of misdiagnosis is considered low [8]. Another limitation is the limited follow‐up of the cases, as most of the cats were managed by their primary care veterinarian and did not return for repeated ophthalmic follow‐up examinations. The timing of follow‐up examinations was inconsistent and was often scheduled depending on pre‐existing visits to the Internal Medicine service and client availability. This was a limiting factor as clinical resolution of uveitis might have occurred sooner than the documented time. The financial burden of remdesivir and GS‐441524 treatment resulted in many owners being unable to pursue further ophthalmic monitoring at the QMHA despite recommendations. In addition, some owners expressed concern that stress, particularly associated with travel, could contribute to FIP relapse, leading to reluctance to return to the QMHA, especially in cases where long distances were required to access the hospital [33, 34]. These factors contributed significantly to the follow‐up of included cases.

Another limitation of the current study is that the potential systemic absorption of topically administered glucocorticoid/NSAIDs via the conjunctiva and nasolacrimal duct was not quantified. As such, we were unable to determine whether systemic corticosteroid/NSAID exposure could have contributed to adverse effects, such as gastrointestinal ulceration, particularly in cats receiving concurrent systemic NSAID therapy. Glucocorticoid administration could also exacerbate clinical disease and reduce capacity for viral clearance in cats with FIP [18, 35, 36].

In conclusion, cats diagnosed with FIP‐associated uveitis have a good prognosis for resolution of ocular inflammation after an 84‐day course of antiviral medication combined with anti‐inflammatory medication. Persistent uveitis and secondary glaucoma can occur so close monitoring of ophthalmic changes is recommended particularly during the early stages of treatment.

## Disclosure

Authors declare no off‐label use of antimicrobials.

## Ethics Statement

Approval was granted for this study by the Social Science Research Ethical Review Board at The Royal Veterinary College (ethical approval URN:SS2021‐0208). Authors declare human ethics approval was not needed.

## Conflicts of Interest

The authors declare no conflicts of interest.

## Supporting information


**Data S1:** jvim70253‐sup‐0001‐NDSRreferenceranges.docx.

**Table S1:** jvim70253‐sup‐0002‐TableS1.xlsx.

**Data S2:** jvim70253‐sup‐0003‐Supinfo.docx.
