# Supplementary material for: Treatment With Remdesivir Alone or in Combination With GS‐441524 in Cats With Ocular Involvement of Feline Infectious Peritonitis: An Observational Case Series
Source: J Vet Intern Med. 2025 Oct 1;39(6):e70253. doi: 10.1111/jvim.70253 (PMC12489177; doi:10.1111/jvim.70253)
Supplement: Supplementary file 1 — Data S1: jvim70253‐sup‐0001‐NDSRreferenceranges.docx. [file JVIM-39-e70253-s002.docx]

Supplementary information

Normal reference ranges for cat 20

| Albumin | 22-39 g/L |
| --- | --- |
| Globulin | 28-48 g/L |
| Bilirubin | 0-15 μmol/L |
| ALT | 12-115 U/L |
| Creatinine | 53-141 μmol/L |
| Neutrophils | 2.3-10.29 x 10^9^/L |
| Lymphocytes | 0.92-6.88 x 10^9^/L |
