# Supplementary material for: Treatment With Remdesivir Alone or in Combination With GS‐441524 in Cats With Ocular Involvement of Feline Infectious Peritonitis: An Observational Case Series
Source: J Vet Intern Med. 2025 Oct 1;39(6):e70253. doi: 10.1111/jvim.70253 (PMC12489177; doi:10.1111/jvim.70253)
Supplement: Supplementary file 3 — Data S2: jvim70253‐sup‐0003‐Supinfo.docx. [file JVIM-39-e70253-s003.docx]

Supplementary information

**Table S1.** Presenting clinical signs, diagnostic tests and outcomes of feline infectious peritonitis cases. Abbreviations: AGP, acid glycoprotein; AU, anterior uveitis; BSH, British short hair; DSH, domestic short hair; FCoV, feline corona virus; FIP, feline infectious peritonitis; FE, female entire; FN, female neutered; FNA, fine needle aspirate; ICC, immunocytochemistry; IHC, immunohistochemistry; LTF, lost to follow-up; m, months; ME, male entire; MN, male neutered; NA, not applicable; NP, not performed; OD, oculus dexter; OS, oculus sinister; PaU, panuveitis; PU, posterior uveitis; PCR, polymerase chain reaction; RT- PCR, reverse transcription polymerase chain reaction; SM, supplementary materials.

Histopathology reports

Cat 4:

Microscopic Diagnoses

1. Anterior chamber; moderate amount of protein-rich fluid with low to moderate

numbers of neutrophils and macrophages

1. Iris, trabecular meshwork, ciliary body, choroid and retina; mild to moderate,

plasmacytic, lymphocytic and histiocytic uveitis, choroiditis and retinitis

3. Iridocorneal (drainage) angle; organised PIFM and marked collapse

4. Iris; posterior synechiae

5. Posterior chamber; moderate haemorrhage with low amounts of fibrin and low

numbers of neutrophils and macrophages

6. Retina; multifocal detachment and inner retinal atrophy

Cat 20:

Microscopic Diagnoses

1. Chronic mild to moderate lymphoplasmacytic anterior (and mild intermediate) uveitis

2. Peripheral anterior synechiae

3. Posterior synechiae

4. Posterior cortical cataract (focal)

5. Glaucoma (secondary)
